# Progress in Diseases of the Blood

**Published:** 1903-04-25

**Authors:** 


### Progress in Diseases of the Blood.

**Thrombosis.**—Considering the large number of persons who come to a premature death, or, at least, enter into a miserably crippled existence, through the occurrence of cerebral hæmorrhage or occlusion, great interest will always be felt in any studies which may lessen the liability to these accidents. Thus Clifford Allbutt's paper on the Causes of High Tension in Later Life and Professor Wright's on Thrombosis in Typhoid open up most important subjects. Thrombosis does not often occur after typhoid in England, but was very common during the African war, whether due to the patients' over-use of their legs or to feeding upon tinned foods. Now Professor Wright[1] finds that the coagulability of the blood and its contents in lime salts varies not only in different individuals but at different times in the same individual. Thus the coagulation time in the male adult varies from $3\frac{1}{2}$ to 15 minutes, and the amount of oxalate of ammonium required to prevent coagulation varies from 1 in 800 to 1 in 2,000, though, indeed, the amount of lime salts present is not always and solely the test of coagulability. In typhoid there is a diminution of coagulability during the early stages which may explain the frequency of intestinal hæmorrhages, but during convalescence there is a great increase. The time taken in clotting in certain cases examined was $4\frac{1}{2}$ times shorter than during the acute stage, and the amount of lime salts present was found to be twice the normal. In four cases where thrombosis occurred the coagulation was abnormally rapid. Therefore it seemed that one cause of thrombosis might be the excess of lime salts, which can be accounted for by the milk diet, for milk contains more lime than ordinary lime water. In other diseases such as Malta fever, where a milk diet is not employed, such thrombosis does not take place. Again, this increase of lime from a milk diet throws light on the value of that diet in acute nephritis with hæmaturia. To prevent its ill effects during convalence from typhoid and to stop the extension of a thrombosis when commenced, he recommends the addition of citrate of soda, 30 grains to the pint of milk, or the juice of two lemons daily. Rolleston suggests that the use of tinned milk in South Africa, with its large percentage of lime salts, may possibly account for the thrombosis in typhoid patients there. It has, however, been thought that phlebitis depends on the action of typhoid bacilli upon the vessel walls, and as to this factor Wright at present has nothing to say. Typhoid gangrene is perhaps even a rarer complication, for only 149 cases are recorded, which seems to depend partly on the tendency of the blood to clot and partly on arteritis, due to the action of the bacilli on the walls of arteries. Nammack[2] mentions two forms, an obliterating arteritis and a parietal one, and adds that the occurrence of gangrene is so rare that no instance was found in 2,000 post-mortems on typhoid cases, though the same series included 59 instances of venous thrombosis. He reports an instance in a patient of his own, a male aged 29, and finds that the most frequent stage at which it appears is the second or third week of the fever, though it may come on as late as the seventh week. The evidence as to the action of the bacilli in this arteritis and in the thrombosis discussed by Wright seems uncertain.

**Raynaud's Disease.**—H. Cushing[3] reports a case marked by arterial spasm and much pain in the upper and lower limbs. A flat rubber bandage was one day applied to the arms for a minute or two and it was found that this was followed by relief from pain and by a temporary vaso-motor relaxation. The bandage was then applied daily to one limb or another and not only did it relieve the burning pain, but it caused a steady improvement in the circulation, so that in six weeks the patient was apparently quite well. The tourniquet seems to temporarily paralyse the vaso-motor nerves so that the terminal arterioles relax and the blood stream can again pass through them.

**Hæmoglobinuria.**—C. Hermann[4] mentions a case in a boy of four years. There was a history of congenital syphilis which, by the way, occurs in half the cases seen in children. The attacks came on at about 10 in the morning every few days, and could be induced by cold baths, over-exertion, or shock. If a ligature was placed around one finger and the hand put into cold water for ten minutes, blood drawn from the finger and allowed to clot gave a red serum, though cold applied to a capillary tube containing blood from other parts of the body produced nothing abnormal. The syphilitic taint should be treated and the possibility of the dovelopment of nephritis must be borne in mind.

**Blood Counts.**—Pneumococcal affections of the various organs such as joints, mucous and serous cavities, have attracted much attention of late, and the blood changes may be important aids to diagnosis. Hewetson's important papers on the blood in pneumonia and appendicitis in the *Birmingham Medical Review*, have been already referred to. A fatal instance of pneumococcal septicæmia in a child with practically normal temperature was reported by G. Parker.[5] The white cells numbered 54,500 and later on 73,000, the Hæomoglobin was 50 per cent., and the red cells over 4,000,000. The lymphocytes numbered 45 per cent., the large hyaline 4·5 per cent., and there were myelocytes both neutrophile and eosinophile, as well as nucleated red cells present. This case illustrates some of the peculiarities of pneumonia which may be seen in children, notably the excess of lymphocytes instead of polynuclear cells, the frequency of large hyaline cells after the acute stage is over, and the presence of eosin myelocytes. Such a large leucocytosis again would be either due to pus, leukæmia, or to some acute infection such as pneumonia, and in this case the diplococcus was found in the heart blood, the peritoneum and the lungs. H. M. King[6] discusses the leucocytosis after operations, and records a series of counts commencing in each case before the operation and carried on until all danger of sepsis had passed away. From these he concludes that an increase of 5,000 to 10,000 leucocytes is normal in the first or second day after operations provided it quickly disappears again. The maximum is usually attained within the first 12 hours, and when the conditions are normal, this increase bears little proportion to the pulse and temperature. An increase of 10,000 or more above the normal state of the individual, if sustained for more than a few hours is suspicious of septic infection. The apparent increase in red cells is not due to an actual increase of the numbers in the circulating blood. *In burns* E. A. Locke[7] records the changes found in 10 cases. The blood was taken from an uninjured part, and was purplish in colour, the red cells were increased by 1,000,000 or 2,000,000, and in fatal cases by even as much as 4,000,000. There was a leucocytosis amounting to about 35,000, and in fatal cases to more than 50,000. This included a slight increase of neutrophile cells, and usually a few myelocytes. A great destruction of leucocytes usually takes place, and an increase of blood-plates occurs. *In lead poisoning*, A. Wolf[8] found the characteristics of this type of anæmia, and a leucocytosis of 25,000, the polymorphonuclear cells being 86 per cent. No myelocytes were present. In the marrow there was a great preponderance of the non-granular over the granular cells and a few normoblasts, while the spleen showed numerous myelocytes and normoblasts, as though it had taken on the work of the marrow.

[1] Med. Rec., Dec. 27, and Brit. Med. Jour., Nov. 29. [2] Med. Rec., Dec. 27. [3] Ibid., Nov. 29. [4] Ibid, Jan. 17. [5] Brit. Med. Jour., March. [6] Amer. Jour. of Med. Sci., Sept. [7] Lancet, Nov. 29. [8] Med. Rec., Oct. 4.

